# The Simon Effect Asymmetry for Left- and Right-Dominant Persons

**DOI:** 10.5334/joc.265

**Published:** 2023-03-03

**Authors:** Robert W. Proctor, Qi Zhong, Jing Chen

**Affiliations:** 1 Dept. of Psychological Sciences, Purdue University, W. Lafayette, IN, US; 2 Dept. of Psychological Sciences, Rice University, Houston, TX, US

**Keywords:** Effector efficiency, Foot responses, Handedness, Simon effect, Simon effect asymmetry

## Abstract

When participants respond to a task-relevant stimulus attribute by pressing a left or right key with the respective index finger, reaction time is shorter if task-irrelevant left-right stimulus location corresponds to that of the response key than if it does not. For right-handers, this Simon effect is larger for right-located than left-located stimuli; for left-handers this Simon-effect asymmetry is reversed. A similar asymmetry has been found for right-footers pressing pedals with their feet. For analyses that separate stimulus- and response-location factors, these asymmetries appear as a main effect of response location, with responses being faster with the dominant effector. If the Simon-effect asymmetry is strictly a function of effector dominance, it should reverse for left-footers responding with their feet. In Experiment 1, left-dominant persons showed faster responses with the left than right hand but with the right than left foot, a finding consistent with prior research on tapping actions. Right-dominant persons also showed the right-foot asymmetry but, unexpectedly, not the typical asymmetry with hand responses. To evaluate whether hand-presses yield results distinct from finger-presses, in Experiment 2 participants performed the Simon task with finger-presses and hand-presses. The opposing asymmetries for right- and left-dominant persons were evident for both response modes. Our results are consistent with the view that the Simon effect asymmetry is primarily due to differences in effector efficiency, usually but not always favoring the dominant effector.

The *Simon effect*, named after J. R. Simon (1990), has been widely used as a tool to investigate various issues in response activation and selection due to the correspondence of variation on a nominally irrelevant stimulus dimension (usually spatial location) with left and right response alternatives (see Hommel, 2011; Lu & Proctor, 1995; Proctor, 2011, for reviews). Participants are typically instructed to make a left or right keypress response to a stimulus feature such as red or green color; the stimulus occurs in a left or right location, which is not relevant to the task. Yet reaction times (RTs) are shorter and response accuracy higher when the stimulus and response locations correspond than when they do not. This benefit of spatial correspondence is often described as “automatic” since it is not an intention as specified in the instructions (e.g., Luo & Proctor, 2022). The most likely reason why the correspondence between stimulus and response locations affects performance is that the left/right discrimination has been defined as relevant to the responses (Ansorge & Wühr, 2004; Yamaguchi & Proctor, 2012).

In the first decade of the 21^st^ century, several studies established that a *Simon effect asymmetry* is evident when left-hand responses are analyzed separately from right-hand responses (Rubichi & Nicoletti, 2006; Spironelli et al., 2009; Tagliabue et al., 2007). For right-handed persons, the Simon effect is larger for the right stimulus location (which corresponds with the right response) than for the left stimulus location (which does not correspond). This relation is reversed for left-handed persons, for which the Simon effect is larger for the left than right stimulus location (Rubichi & Nicoletti, 2006; Tagliabue et al. 2007). These and other findings led the authors of the three studies to propose that the Simon effect asymmetry is due to an attentional bias that favors the operating space of the dominant effector. For right-handers, this bias produces stronger coding of the stimulus location in the right visual hemifield, whereas for left-handers it produces stronger coding of the stimulus location in the left visual hemifield.

There are two ways to analyze the performance data in a Simon task, due to the fact that correspondence on a trial is determined by the relation between the stimulus location and response location on that trial. One common way to code data from a Simon task in an analysis of variance (ANOVA) is with correspondence (C: noncorresponding vs. corresponding) as a factor rather than separating stimulus location and response location (e.g., Proctor & Lu, 1999; Tagliabue et al., 2000). This coding simplifies interpretation of the results because the Simon effect is shown by a main effect of correspondence and its interaction with other variables can be easily evaluated. Rubichi and Nicoletti (2006) performed such a correspondence ANOVA, shown in the left column of Table 1, including the additional factors of stimulus location (StL: left vs. right) and handedness (H: left-handed vs. right-handed). In that type of ANOVA, the C main effect indicates an overall Simon effect and the asymmetry is indicated by the C × StL interaction. Accordingly, a difference in the asymmetry for left- and right-handed persons shows up as the 3-way interaction C × StL × H, which was significant in Rubichi and Nicoletti’s study.

**Table 1:** Comparison of Two Different ANOVAs for Evaluating the Simon Effect Asymmetry.


	CORRESPONDENCE ANOVA	STL × REL ANOVA

1	StL	StL

2	StL × C	ReL

3	StL × H	StL × H

4	StL × C × H	ReL × H

5	C	StL × ReL

6	C × H	StL × ReL × H


*Note*: Stimulus Location = StL; Response Location = ReL, Correspondence = C, and Handedness = H. Because the two ANOVAs are performed on the same data set, just partitioning them differently, the two terms on each row yield the same *F* ratios.

A limitation of using correspondence as a variable when evaluating whether the Simon effect is asymmetric is that the C × StL interaction includes a subtle confound. That is, the Simon effect for the left stimulus (St_left_) is calculated by subtracting RT for the left response (Re_left_) from RT for the right response (Re_right_), whereas the Simon effect for the right stimulus (St_right_) is calculated by subtracting RT for the right response (Re_right_) from RT for the left response (Re_left_). Thus, the dominant effector provides the corresponding response for one stimulus location but the noncorresponding response for the other, which may cause an asymmetry in the data that has nothing to do with correspondence. Consequently, the apparent asymmetry in Simon effects between left and right stimulus locations could instead be due to the difference between the response hands/locations (faster right than left responses for right-handed persons; faster left than right responses for left-handed persons).

An alternative way to analyze the same data from a Simon effect experiment is to code stimulus location (StL: St_left_ vs. St_right_) and response location (ReL: Re_left_ vs. Re_right_) as separate factors, instead of using correspondence as a factor. Tagliabue et al. (2007) pointed out that an ANOVA of the data coded in this manner, with handedness as a third factor, yields the same *F* ratios as the correspondence ANOVA, just with the relation to the factors changed. With the StL × ReL coding, the Simon effect is indicated by a significant interaction of stimulus location and response location rather than by a C main effect (compare the entries on row 5 of Table 1), with the resulting *F* ratios being identical. This equivalence of different terms holds true across the entire ANOVA produced using StL × ReL coding vs. correspondence coding: The same *F* ratios are output for the data set, but with the terms rearranged (as shown for all rows in Table 1).

Of importance, the C × StL interaction in the correspondence ANOVA, interpreted as a larger Simon effect for one of the two stimulus locations, shows up as a ReL main effect in the StL × ReL ANOVA (row 2). That is, this ANOVA shows that the apparent asymmetry in the Simon effect actually reflects faster responding with one hand than the other. Accordingly, the 3-way interaction of StL × C × H in the correspondence ANOVA is the two-way interaction term of ReL × H in the alternative ANOVA, reflecting faster responses with the dominant hand. In other words, because the measure of the Simon effect when stimulus and response locations are coded separately, the StL × ReL interaction, collapses across stimulus and response locations, it is not confounded with either, and the apparent interaction of the Simon effect with stimulus location is revealed to be a main effect of response location (or hand) that may interact with handedness. Note also that (a) even when there is no significant overall StL × ReL interaction, the response location main effect still reflects an asymmetry (opposing Simon effects for the left and right stimulus locations) and (b) the terms representing the stimulus location main effect and the stimulus location × handedness interaction do not change across the two ANOVAs, meaning that those terms are unrelated to the Simon effect.

Seibold et al. (2016) replicated the interaction of the Simon effect asymmetry with handedness found in the earlier studies, although their left-handed participants did not show a complete reversal to a larger effect for the left stimulus location. However, a StL × ReL ANOVA showed that the result was due to the following: Right-handers showed the Simon effect asymmetry favoring the right stimulus location, which was due to their responses being faster overall with the right than the left hand (i.e., a difference in effector efficiency for left and right effectors). Left-handers’ responses showed a nonsignificant opposite tendency, indicating that they did not show the benefit for right responses that the right-handers did.

Although less publicized than handedness, people also have a dominant foot used to perform manipulative actions (like kicking a ball), while the non-dominant foot provides stability (Gabbard & Hart, 1996). Foot dominance is highly correlated with hand dominance (e.g., Peters & Durding, 1979), although it is not quite as strong (i.e., there is more mixed footedness – i.e., no consistent dominance of the right or left foot – than mixed handedness; Tran et al., 2014). Foot-press responses are rarely used in studies of Simon-type tasks. One exception is a study by Proctor et al. (2003) which showed that a left-right Simon effect was obtained with stimuli that also varied along a vertical location dimension when a foot response was paired with a hand response. Another more relevant exception is a study of Phillips and Ward (2002, Experiment 3), which showed correspondence effects with left and right foot-press responses for the side to which object handles were oriented. However, since their analysis did not report the results separately for left and right feet, whether the Simon effect asymmetry was evident for foot-responses cannot be determined. Although not testing the Simon effect, Peters and Durding (1979) demonstrated that tapping responses can be made faster with the right foot than with the left foot for right-dominant persons. Thus, the effector efficiency hypothesis predicts that right-dominant persons should show a Simon effect asymmetry favoring the right stimulus location (i.e., dominant right-hand response) over the left location.

This prediction was tested by Chen et al. (2021) in a study that included only right-dominant persons (due to their availability). Chen et al.’s Experiment 1 demonstrated that pedal-press responses made with the left and right feet yielded a Simon effect asymmetry favoring the right response, like that obtained with the index fingers of the left and right hands. Experiments 2 and 3 showed that the Simon effect asymmetry for RT was evident even when the participants were unable to see the response device and their effectors while responding with the left and right feet or the index fingers. Although both the hands and feet showed a Simon effect asymmetry in RT, the pattern of incorrect responses (a preponderance of errors being incorrectly responding with the right foot on noncorresponding trials for which the left response would be correct) suggested that the asymmetry for the feet could be due at least in part to a bias to make the dominant response, whereas the asymmetry for hands is not due to such response bias. Regardless, for both response modes, the results suggested that, for right-dominant persons, the Simon effect asymmetry is mainly a consequence of comparing conditions for which one response is made by the faster dominant right effector and the other with the slower left effector.

The prediction for left-dominant persons is less clear-cut. Peters and Durding (1979) found that although right-handed persons made tapping responses faster with their right hand and right foot, left-handed persons (the majority of whom were also left-foot dominant) were faster tapping with the left hand but the right foot. Strictly based on effector dominance, as determined by answers to foot-preference questions, the Simon effect for left-dominant persons would be expected to favor the left foot. However, Peters and Durding’s results for tapping responses suggest that left-dominant persons may be more efficient pressing a pedal with the right foot. Thus, effector efficiency, instead of the nominal effector dominance based on subjective responses to questionnaires, may be a better indicator of the Simon effect asymmetry.

A general property of the Simon effect with left and right keypress responses is that it decreases across the RT distribution (De Jong et al., 1994; Proctor et al., 2011). Separate RT bin analyses of the Simon effect and the dominant-hand advantage across the RT distribution have found that for finger-press responses the dominant-hand advantage does not follow the time-course of the Simon effect, remaining flat or even increasing slightly across the RT distribution, suggesting that different processes underlie each (Rubichi & Nicoletti, 2006; Seibold et al., 2016). Such analyses have not been reported previously for foot-press or hand-press responses on pedals. If the difference in time courses is evident for those response modes as well, it will provide additional evidence that the effect of effector efficiency is distinct from the Simon effect.

To summarize, the present experiments were designed to address several issues that remained unresolved after Chen et al.’s (2021) study:

1. Because left-dominant persons were not tested by Chen et al., whether foot-press responses show the reversal of the Simon effect asymmetry predicted based on effector dominance remained unanswered. This void was filled in the present study by testing both left- and right-dominant persons.
2. Because the hand responses were made with presses of the index fingers on the outer two keys of a five-row response box and the foot responses with presses of the whole foot on driver-simulator gas pedals, the response keys and forces required to operate them were quite different. These differences were minimized in the present Experiment 1 by having the hand responses made with palm presses on the same pedals as the foot responses.
3. Due to said differences, the temporal dynamics of the Simon effect and its asymmetry across the RT distribution could not be examined meaningfully in Chen et al.’s study. The designs of the present experiments enabled comparison of the effects obtained with the respective response modes using delta plots of error rate and RT for measures of the Simon effect and its asymmetry across 20-percentile bins (Proctor et al., 2011).
4. Because, unlike prior findings with index-finger responses, the hand responses for the right-dominant persons did not show a significant Simon effect asymmetry in the present Experiment 1, Experiment 2 compared performance with hand presses of the pedals to that with finger presses on response-box keys.

## Experiment 1: Hand Presses on Pedals vs. Foot Presses on Pedals

Experiment 1 evaluated the Simon effect and its asymmetry for left- and right-dominant persons. Hand- and foot-press responses were made by each participant in distinct trial blocks. The effector efficiency hypothesis predicts that right-dominant persons should show an advantage for the right response for both hand- and foot-press responses; left-dominant persons should show an advantage for the left hand-press response but, given Peters and Durding’s (1979) findings for tapping responses, possibly an advantage for the right foot-press response.

### Method

Left-handed persons make up only about 10% of the population (de Kovel et al., 2019), which limits their availability as research participants. Prior studies included 20 and 16 participants per hand-dominance group (Rubichi et al., 2006, Experiments 1 and 2), 12 per group (Tagliabue et al., 2007, Experiment 2), and 24 per group (Seibold et al., 2016, Experiment 1). These numbers have been adequate to show the Simon effect and its asymmetry in all cases. In the present study, another limitation was that persons needed to be left- or right-dominant for both handedness and footedness, which restricted the eligible participants further (because the two do not correlate perfectly and mixed preference is more common for footedness; Tran et al., 2014). Thus, we tried to maximize the number of left-dominant persons available at the time and run a similar number of right-dominant persons. Also, to maximize sensitivity for detecting response mode effects, we manipulated responding with the hands or feet within-subjects.

#### Participants

Sixty-five undergraduates from introductory psychology classes at Purdue University participated for credit toward a course requirement. Preferences for the left or right hand were obtained from subjective responses to the Edinburgh Handedness Inventory (Oldfield, 1971), and preferences for the left or right foot were obtained from the Waterloo Footedness Questionnaire – Revised (Elias et al., 1998). Thirty-one participants were left-dominant (16 female, 15 male; mean age = 19.7 years, *SD* = 1.7 years): mean handedness score –87.02 ± 15.15 (on a scale of –100 to +100), and mean footedness score –9.23 ± 4.00 [total of answers to 10 questions of left always (–2), left usually (–1), equally often (0), right usually (+1), right always (+2)]. Thirty-four were right-dominant (22 female, 12 male; mean age = 18.6 years, *SD* = 1.0 years): mean handedness score 90.38 ± 9.23 and mean footedness score 10.63 ± 5.11.

#### Apparatus, Stimuli, and Procedure

**Figure 1:**
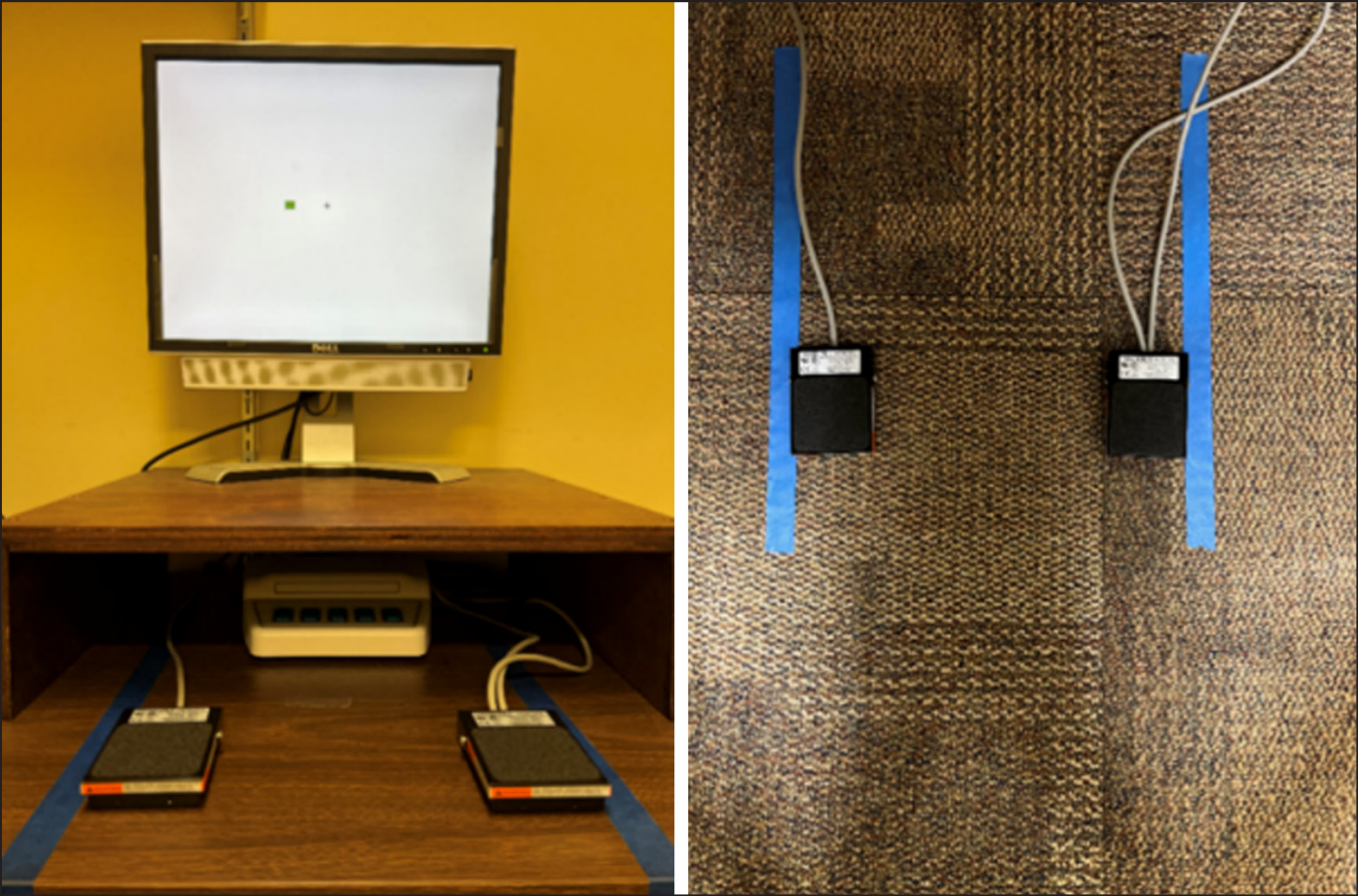
Display Screen and Pedals on Table (Left Panel) and on Floor (Right Panel).

The experiment was conducted on a Dell Optiplex 745 personal computer with a 19-in. LCD color monitor, set on top of a wood-frame box (see Figure 1). Stimulus presentation, response recording, and feedback were controlled by E-prime 2.0 software. Stimuli were red and green rectangles of 1.1° × 0.9° visual angle, presented 3.8° to the left or right of a centered fixation cross (0.7° × 0.7°), displayed on a white background. Responses were made by pressing a left or right Treadlite II foot pedal, placed on the tabletop (hand-press condition; see Figure 1, left) or floor (foot-press condition; see Figure 1, right).

Each participant performed the Simon task for 12 practice trials and 120 experimental trials in each of two trial blocks, responding with the hands in one block and the feet in the other. The order of the response modes was approximately counterbalanced; within each trial block the stimulus colors and spatial locations occurred in a pseudo-random order, with equal numbers of corresponding and noncorresponding trials. Participants were seated in front of the monitor at about 60 cm and responded by pressing the left or right pedal. With the feet, a response was made by pressing downward with the front half of the foot, like pressing a gas pedal. With the hands, a response was made by a downward motion of the part of the palm near the wrist. This manner of responding was necessary to generate sufficient force to operate the pedal.

Each trial started with a 1,100-ms display of the fixation cross. The stimulus then appeared and stayed on the screen until a response was made or disappeared after 1,500 ms if there was no response. Following the response, a 500-ms feedback screen appeared that showed just the fixation cross for correct responses or the word “Error” below the fixation cross for incorrect responses.

#### Design

The independent variables were stimulus location (left vs. right), response location (left vs. right), and response mode (hands or feet), all within-subjects, and effector dominance (left-dominant vs. right-dominant), which was between-subjects. The dependent variables were RT and percentage error (PE). Separate ANOVAs were performed on the RT and PE data.

### Results

#### Reaction Time

**Figure 2:**
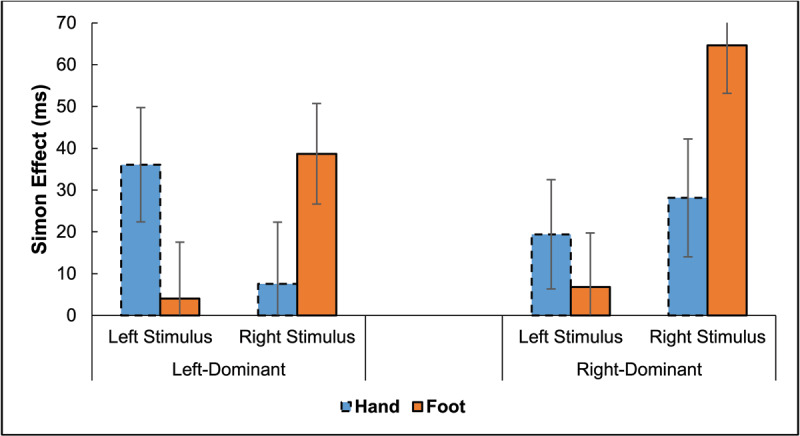
Experiment 1: Simon Effect in Reaction Times (RTs) as a Function of Stimulus Location and Response Mode (Hands, Feet). *Note*: Error bars represent 95% confidence intervals.

Error trials (2.5%) and trials with RT > 900 ms or < 100 ms (1.0%) were excluded from the RT analysis. For clarity, Figure 2 plots the Simon effect as a function of stimulus location and hand dominance. It shows overall Simon effects for both response modes and asymmetries of the Simon effect favoring the stimulus location corresponding with the dominant hand, except for foot responses of left-dominant participants. Recollect, though, this method of calculation of the Simon effect for the respective stimulus locations includes a confound with hand differences.

**Figure 3:**
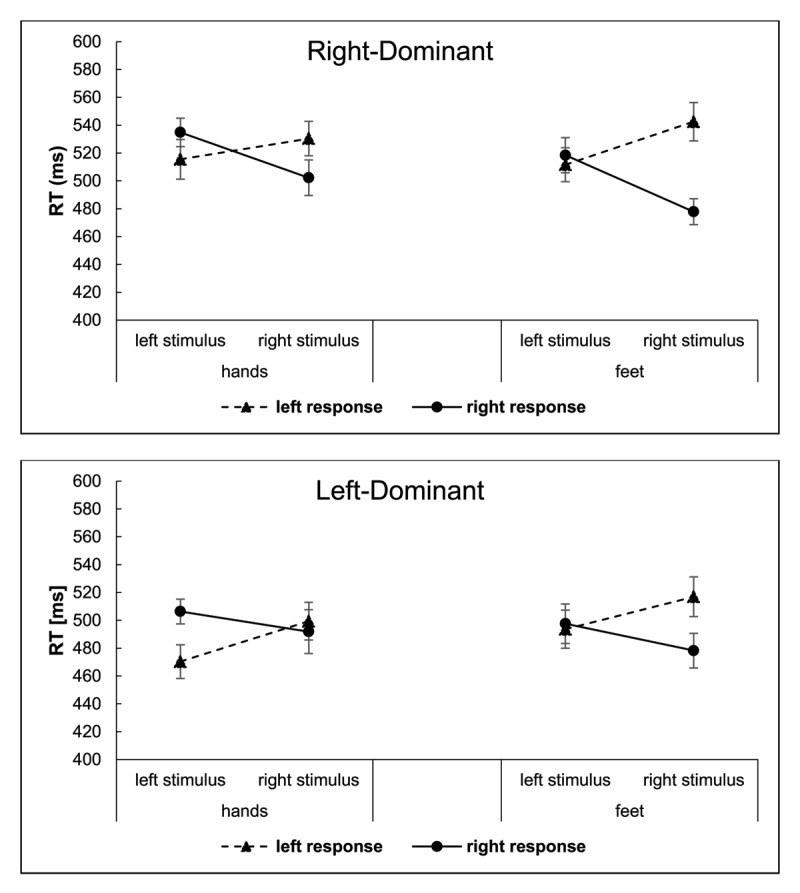
Experiment 1: Reaction Times (RTs) for Right-Dominant (Top) and Left-Dominant (Bottom) Persons as Functions of Stimulus and Response Locations, and Response Mode (Hands, Feet). *Note*: The error bars reflect Cousineau-Morey 95% confidence intervals (Morey, 2008).

We report the results of a StL × ReL ANOVA that distinguishes stimulus and response locations, as well as effector dominance and response mode, because it does not contain that confound. The mean data from this ANOVA are portrayed in Figure 3. Results showed a stimulus location × response location interaction, *F*(1, 63) = 103.25, *p* < .001, \eta _p^2 = .62, indicating a Simon effect of 26 ms, and a main effect of response location, *F*(1, 63) = 8.09, *p* = .006, \eta _p^2 = .11, reflecting an overall Simon effect asymmetry (right responses 9 ms < left responses; Seibold et al., 2016). This effect of response location interacted with response mode, *F*(1, 63) = 26.38, *p* < .001, \eta _p^2 = .30: RT was shorter for right foot-press responses than for left foot-press responses (*M*s = 493 vs. 516 ms), *p* < .001, but there was no significant difference between the left and right hand-press responses (*M*s = 504 vs. 509 ms), *p* = .273.

Of primary interest was effector dominance, for which the main effect was not significant, *F*(1, 63) = 2.08, *p* = .155, \eta _p^2 = .03. The interaction of effector dominance with stimulus location and response location was also nonsignificant, *F*(1, 63) = 2.62, *p* = .111, \eta _p^2 = .04, indicating that the Simon effect did not differ reliably between the two effector dominance groups. Effector dominance did interact with response location, *F*(1, 63) = 5.61, *p* = .021, \eta _p^2 = .08: The response-location effect was significant for the right-dominant group (*M*s = 525 ms for left responses vs. 508 ms for right responses), *F*(1, 33) = 11.54, *p* = .002, \eta _p^2 = .26, but not the left-dominant group, *F* < 1.0 (*M*s = 495 ms for left responses vs. 494 ms for right responses). Another way to describe this result, thus, is that the right-dominant group showed a significant Simon effect asymmetry, but the left-dominant group did not.

Separate analyses of the respective effector dominance groups showed the following. For right-dominant persons, response mode entered into a 2-way interaction with response location *F*(1, 33) = 8.30, *p* = .007, \eta _p^2 = .20, reflected by a significant difference in response location for the foot responses, *p* < .001, but not for the hand responses, *p* = .537. Response mode also entered a 3-way interaction with stimulus location and response location *F*(1, 33) = 5.38, *p* = .027, \eta _p^2 = .14. As shown in Figure 3, these reflect that the Simon effect asymmetry and the Simon effect itself were larger with foot presses than with hand presses for the right-dominant persons. The left-dominant group also showed a significant interaction of response mode × response location, *F*(1, 30) = 22.84, *p* < .001, \eta _p^2 = .43, but not the 3-way interaction, *F* < 1.0. The left hand-presses were faster than the right hand-presses (*M*s = 485 ms vs. 499 ms), *p* = .012, but the right foot-responses were faster than the left foot-presses (*M*s = 488 ms vs. 505 ms), *p* = .002. Thus, even though the participants in the latter group were classified as left-dominant by their foot preferences, their RT results were like those for right-foot dominant persons.

Returning to the overall ANOVA, effector dominance also interacted with stimulus location, *F*(1, 63) = 9.34, *p* = .003, \eta _p^2 = .13: Right stimuli were responded to faster than left stimuli for the right-dominant group (*M*s = 513 vs. 520 ms), *p* = .010, but there was no significant difference for the left-dominant group (*M*s = 492 ms for left stimuli vs. 497 ms for right stimuli), *p* = .095. No other effect was significant, *p*s > .05.

#### Percentage Error

**Figure 4:**
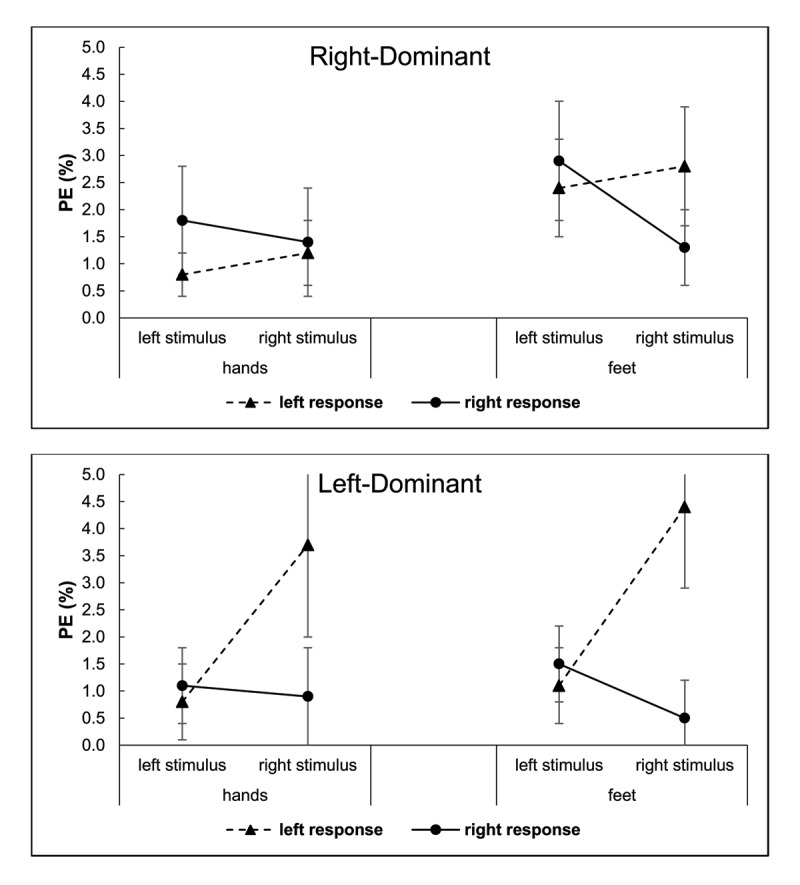
Experiment 1: Percentage Error (PE) for Right-Dominant (Top) and Left-Dominant (Bottom) Persons as a Function of Stimulus and Response Locations, and Response Mode (Hands, Feet). *Note*: The error bars reflect Cousineau-Morey 95% confidence intervals (Morey, 2008).

A similar overall ANOVA of PE including all independent variables was conducted. The stimulus location × response location interaction was significant, *F*(1, 63) = 23.09, *p* < .001, \eta _p^2 = .27, reflecting a Simon effect in the PE data (see Figure 4). The 3-way interaction of those variables with effector dominance was also significant, *F*(1, 63) = 4.86, *p* = .031, \eta _p^2 = .07. The PE Simon effect was larger for the left-dominant group than for the right-dominant group, but it was still significant for the latter group, *F*(1, 33) = 4.50, *p* = .042, \eta _p^2 = .12. The main effect of response location was significant, *F*(1, 63) = 7.56, *p* = .008, \eta _p^2 = .11, signaling a Simon effect asymmetry overall. This effect of response location was modulated by effector dominance, *F*(1, 63) = 8.51, *p* = .005, \eta _p^2 = .12. Left responses had a higher PE than right responses for the left-dominant group (*M*s = 2.5% vs. 1.0%), *F*(1, 30) = 20.79, *p* < .001, \eta _p^2 = .41. There was no significant difference for the right-dominant group (*M*s = 1.8% for left responses vs. 1.9% for right responses), *F* < 1.0, but that group showed a significant interaction of response mode with response location, *F*(1, 30) = 4.98, *p* = .042, \eta _p^2 = .13. The same interaction was also at the .05 criterion in the overall ANOVA, *F*(1, 63) = 3.99, *p* = .050, \eta _p^2 = .06.

The analysis also showed a main effect of response mode, *F*(1, 63) = 6.45, *p* = .014, \eta _p^2 = .09. Hand-press responses had a lower PE than foot-press responses (*M*s = 1.5% vs. 2.1%). The interaction between stimulus location and effector dominance was also significant, *F*(1, 63) = 9.25, *p* = .003, \eta _p^2 = .13. Right stimuli had a higher PE than left stimuli for the left-dominant group (*M*s = 2.4% vs. 1.1%), *p* = .001, but there was no significant difference for the right-dominant group (*M*s = 2.0% for left stimuli vs. 1.7% for right stimuli), *p* = .364. No other effect was significant, *p*s > .05.

#### Distribution Analyses

We describe the procedure for the PE bin analysis first because it was performed without omitting trials, whereas the RT bin analysis described after it was performed with error and outlier trials omitted. However, when reporting the results, we report the RT bin analysis first to be consistent with reporting of the main analyses.

All RTs were rank ordered including error and outlier trials per condition for each participant, from shortest to longest. To obtain the Simon effect for each bin, the ranking was conducted for the congruent and incongruent trials separately for each response mode (hand vs. foot). To obtain the response location effect (i.e., the difference in Simon effect for dominant and nondominant effectors), the ranking was conducted for the left and right response locations separately for each effector condition (hand vs. foot). After the trials were ranked by RT, they were equally divided into 5 bins, and PE was calculated for each bin. The Simon effect for each bin was then computed by subtracting the bin mean for congruent trials from that for incongruent trials. Similarly, the response location effect for each bin was calculated by subtracting the bin mean for the dominant trials from that for the non-dominant trials. Separate ANOVAs were conducted for the Simon effect and response location effect, with bin (1 through 5) and response mode (hand vs. foot) being within-subjects.

RT bin analysis was done like the PE analysis, except that error and outlier trials outside [100 – 900 ms] were omitted before ranking the trials and dividing them into bins.

##### Reaction Time

**Figure 5:**
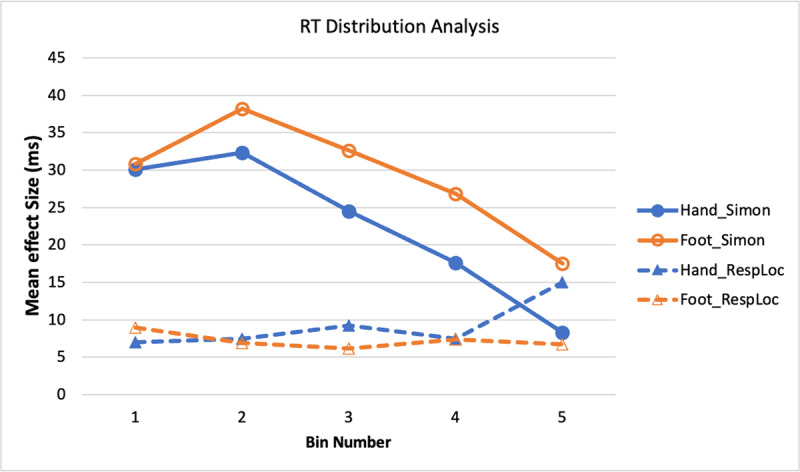
Experiment 1: RT Simon and Response Location Effects as a Function of RT Bin.

Figure 5 shows the distribution functions for the Simon and response-location effects in RT. The most obvious patterns from Figure 5 are: The RT Simon and response-location effects show different time courses, with the Simon effect decreasing across bins 2–5 and the response-location effect being relatively constant. Analysis of the Simon effect showed only a bin main effect, *F*(1.9, 122.3) = 13.17, *p* < .001, \eta _p^2 = .17 (Huynh-Feldt adjustment applied due to violation of sphericity assumption): The result pattern shows the typical finding of the Simon effect being largest early in the RT distribution and then decreasing. Although the Simon effect tended to be larger with the feet than the hands, the main effect was not significant, *F*(1, 64) = 2.54, *p* = .116, \eta _p^2 = .04, nor was the interaction, *F* < 1.0. The similar time course for the hand and foot responses indicates that the Simon effect resides in response-selection processes separate from the effectors.

The response-location effect also showed no significant factors, *F*s < 1.0. The relative lack of change in the response-location effect across the RT distribution, compared to the decreasing function for the Simon effect, implies that the two effects are due to different factors.

##### Percentage Error

**Figure 6:**
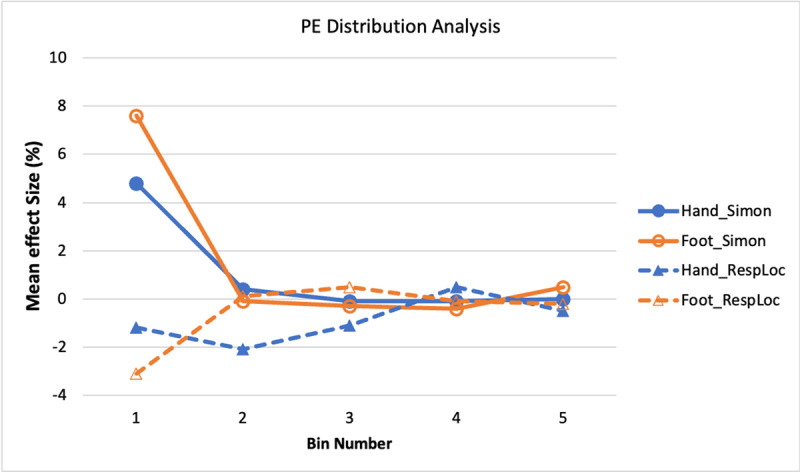
Experiment 1: PE Simon and Response-Location Effects as a Function of RT Bin.

Figure 6 shows the distribution functions for the Simon and response-location effects in PE. The most notable feature of Figure 6 is that the Simon effect functions (solid lines) show more errors in the first bin than in the others, as verified by a bin main effect, *F*(2.1, 133.6) = 19.28, *p* < .001, \eta _p^2 = .23 (Huynh-Feldt adjustment applied). Neither a response mode main effect nor interaction was significant, *p*s > .10. This result pattern indicates a tendency to make the response corresponding to the stimulus location incorrectly on incongruent trials when responding quickly, regardless of whether responding with hands or feet. It thus provides confirming evidence that the Simon effect has a similar basis for the two response modes.

The functions for the response-location effect (dashed lines) also show the most errors at the early bins, though the pattern is not as strong, as indicated by a nonsignificant bin main effect, *F*(2.8, 181.6) = 2.34, *p* = .078, \eta _p^2 = .04 (Huynh-Feldt adjustment applied). The largest effects were at the first and second bins, and the numerical values are negative, indicating a predominance of errors of responding with the non-dominant effector when the correct response should be the dominant one. The functions show no overall difference between feet and hands, *F* < 1.0, but the interaction of bin and response mode was significant, *F*(3.4, 219.4) = 2.87, *p* = .031, \eta _p^2 = .04. (Huynh-Feldt adjustment applied). This interaction is due mainly to the response-location effect being restricted primarily to bin 1 for the foot-press responses but lasting through bins 1–3 for the hand-press responses.

### Discussion

As expected, a Simon effect was obtained for both hand- and foot-press responses and for left- and right-dominant persons. Of more interest was the Simon effect asymmetry, as reflected in the response location main effect, which was evident in the RT data. For hand-presses, the left-dominant group showed the expected shorter RT for the left response than for the right response. However, the right-dominant group did not show a significant asymmetry of shorter RT for the right response than the left response, as has been found in several other studies. This nonsignificant effect is addressed in Experiment 2. For foot-presses, not only the right-dominant group but also the left-dominant group showed an RT advantage for the right response. These results for hand- and foot-press responses agree with those obtained by Peters and Durding (1979) for tapping responses. This deviation of performance efficiency with responses requiring foot presses from the foot preferences expressed by left-footed persons on the footedness questionnaire suggests that there may be a unique property to press-types of movements made with the feet. We discuss this issue more in the General Discussion.

The error data for the foot responses showed an asymmetry of larger Simon effect for the right stimulus position than for the left stimulus position for both right- and left-dominant groups. In both cases, the PE was high when the stimulus appeared in the right location but required a left response. The hand responses showed a lack of significant asymmetry in the PE data as in the RT data for the right-dominant persons, but an asymmetry favoring the right stimulus location for the left-dominant persons, which is counter to the RT data.

For the bin analyses, the PE data showed that the Simon effect was strongest when responses were fast (i.e., in the first bin). This tendency for errors to be the fast responses was not evident in the Simon effect asymmetry, for which the analysis did not show a significant bin main effect. In the RT data, the Simon effect showed the typical pattern of peaking early in the RT distribution and then decreasing across the remainder of it, and this effect was evident for both response modes. The Simon effect asymmetry was relatively flat across the RT distribution, and again, this pattern was similar for both the hand- and foot-press responses. The different distribution patterns for the Simon effect and the asymmetry imply that they have distinct bases.

## Experiment 2: Hand-Presses on Pedals vs. Finger-Presses on Response Box

In Experiment 1, left-dominant persons showed a Simon effect asymmetry in RT for hand-press responses that favored the left hand. However, right-dominant persons did not show the opposite RT asymmetry favoring the right hand, although their foot-press responses did show an asymmetry favoring the right foot. Because the right-hand advantage for right-dominant persons has been obtained reliably when press responses were made with the index fingers, rather than the palms, we deemed it necessary to re-run the hand-press condition, along with a finger-press response condition, in Experiment 2. Direct comparison of those conditions allowed us to evaluate whether the advantage for the right hand is less strong with hand presses than with finger presses, or whether the relation between the Simon effect asymmetry and dominant hand is distinct from how the responses with the left and right hands are implemented.

### Method

#### Participants

A total of 77 new students from the same subject pool as in Experiment 1 took part for credit toward their course requirement. Handedness and footedness were measured as in Experiment 1. Thirty-three were left-handed (22 female, 11 male; mean age = 18.8 years, *SD* = 1.1 years), with a mean handedness score of –82.41 ± 16.47 and mean footedness score of –9.00 ± 5.41. Forty-four were right-handed (26 female, 18 male; mean age = 18.7 years, *SD* = 0.9 years) with mean handedness score of 87.05 ± 15.63 and mean footedness score of 10.02 ± 5.00.

Participants performed two trial blocks, as in Experiment 1, responding in one block with the palms of the hands on the pedals, like in that experiment, and in the other block with the index fingers on the outermost buttons of a row of five on a response box. Otherwise, the method was like Experiment 1. The independent variables were stimulus location (left vs. right; within-participants), response location (left vs. right; within-participants), response mode (finger vs. hand), and dominance (left vs. right). The dependent variables again were RT and PE.

### Results

#### Reaction Time

**Figure 7:**
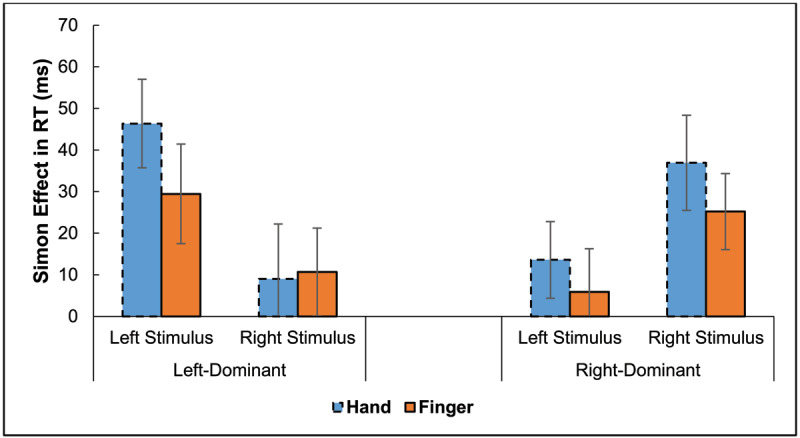
Experiment 2: Simon Effect in RT as a Function of Stimulus Location (Left, Right) and Response Mode (Hands, Fingers). *Note*: The error bars reflect Cousineau-Morey 95% confidence intervals (Morey, 2008).

Error trials (2.3%), as well as trials with RT > 900 ms and < 100 ms (1.8%), were again excluded from the RT analysis. As before, the Simon effect is plotted as a function of stimulus location (see Figure 7), illustrating that both the Simon effect is evident and that its asymmetry is opposite for left- and right-dominant persons but in opposite directions.

The overall ANOVA that partitioned correspondence into stimulus location and response location showed a main effect of response mode, *F*(1, 75) = 229.46, *p* < .001, \eta _p^2 = .75, with the finger responses being faster than the hand responses (*M*s = 421 vs. 497 ms). This difference was likely due at least in part to less force being needed to operate the buttons than the pedals. The two-way interaction of stimulus location x response location was significant, *F*(1, 75) = 106.74, *p* < .001, \eta _p^2 = .59, indicating a Simon effect of 22 ms, which did not interact with effector dominance, *F* < 1.0. However, response mode did interact with stimulus location and response location, *F*(1, 75) = 6.31, *p* = .014, \eta _p^2 = .08: The Simon effect was larger for hand-presses (26 ms) than for finger-presses (18 ms), although both effects were significant separately, *p*s < .001.

**Figure 8:**
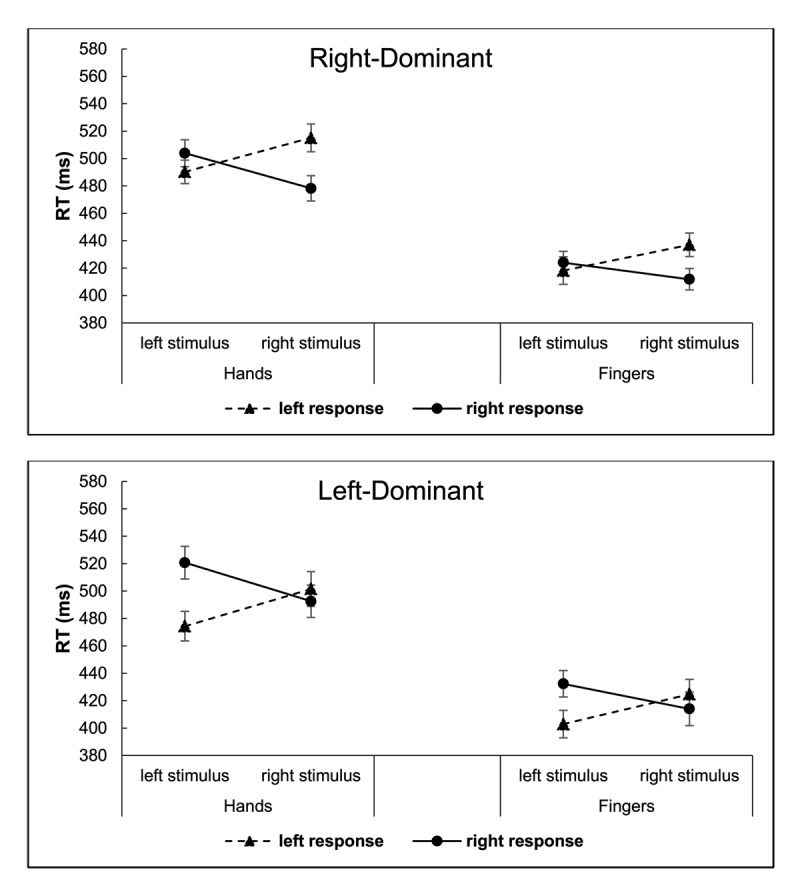
Experiment 2: RT for Right-Dominant (Top) and Left-Dominant (Bottom) Persons as a Function of Stimulus and Response Locations, and Response Mode (Hands, Fingers). *Note*: The error bars reflect Cousineau-Morey 95% confidence intervals (Morey, 2008).

There was no main effect of response location, *F* < 1.0, but there was a response location x effector dominance interaction, *F*(1, 75) = 21.99, *p* < .001, \eta _p^2 = .23. For the right-dominant group, right responses were faster than left responses (*M*s = 454 vs. 465 ms), whereas for the left-dominant group, left responses were faster than right responses (*M*s = 451 vs. 465 ms). Separate ANOVAs for each group showed that the response-location effects were significant for each, *F*(1, 43) = 9.08, *p* = .004, \eta _p^2 = .17, and *F*(1, 32) = 13.38, *p* < .001, \eta _p^2 = .30, respectively. These response effects indicate opposing Simon effect asymmetries, with the right-dominant group showing a larger effect for the right stimulus location and the left-dominant group showing a larger effect for the left stimulus location (see Figure 8).

Both the right- and left-dominant groups showed a significant 2-way interaction of stimulus location and response location, *F*(1, 43) = 117.29, *p* < .001, \eta _p^2 = .73, and *F*(1, 32) = 31.24, *p* < .001, \eta _p^2 = .49, respectively, indicating the Simon effect. The 3-way interaction of those variables with response mode was significant for the right-dominant group *F*(1, 43) = 5.59, *p* = .023, \eta _p^2 = .12, but not the left-dominant group, *F*(1, 32) = 1.73, *p* = .198, \eta _p^2 = .05. However, the mean data showed a larger Simon effect for the hand responses than the finger responses for both.

#### Percentage Error

The two-way interaction between stimulus location and response location was significant, *F*(1, 75) = 4.71, *p* = .033, \eta _p^2 = .06, indicating a Simon effect in PE. The Simon effect was 1.0% for left stimuli, *p* = .009, and 0.1% for right stimuli, *p* = .630. The Simon effect was also modulated by the response mode, indicated by a three-way interaction between response mode, stimulus location, and response location, *F*(1, 75) = 4.12, *p* = .046, \eta _p^2 = .05. The Simon effect was –0.3% for hand responses, *p* = .608, and 2.5% for finger responses, *p* = .028.

There was a main effect of response location, *F*(1, 75) = 4.22, *p* = .043, \eta _p^2 = .05, with PE less for left responses than right responses (*M*s = 2.0% vs. 2.4%). The ANOVA also showed a main effect of response mode, *F*(1, 75) = 47.48, *p* < .001, \eta _p^2 = .39, with PE higher for finger responses than hand responses (*M*s = 3.3% vs. 1.1%). No other effect was significant, *p*s > .05.

**Figure 9:**
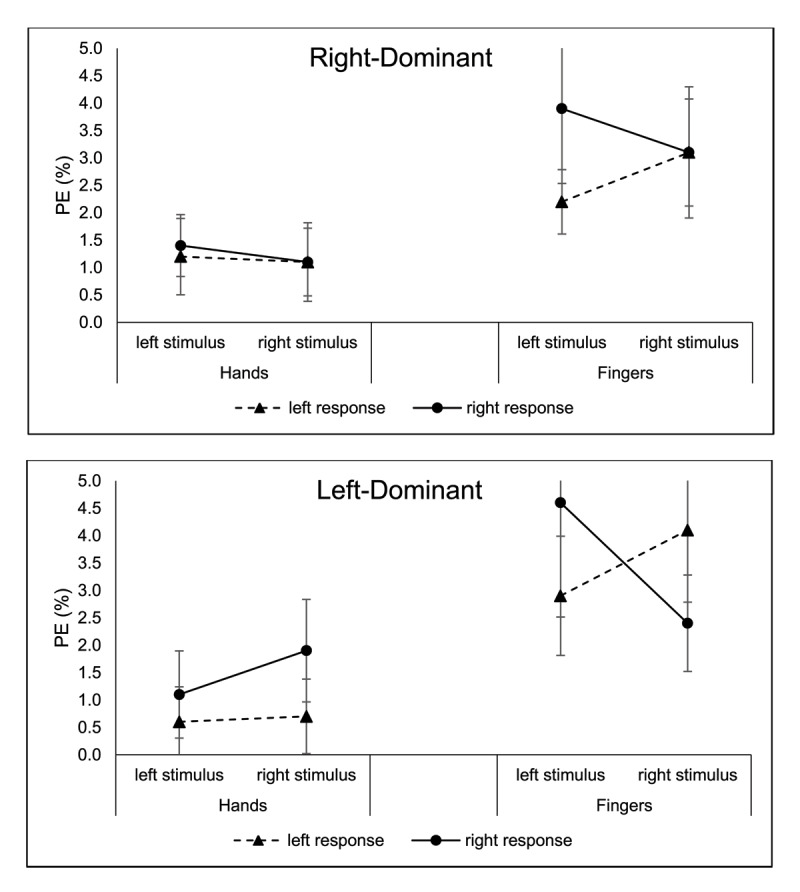
Experiment 2: PE for Right-Dominant (Top) and Left-Dominant (Bottom) Groups as a Function of Stimulus (Left, Right) and Response Locations (Left, Right), and Response Mode (Hands, Fingers). *Note*: The error bars reflect Cousineau-Morey 95% confidence intervals (Morey, 2008).

The right-dominant group showed only a main effect of response mode, *F*(1, 43) = 18.92, *p* < .001, \eta _p^2 = .31, with PE less with the hands (1.2%) than with the fingers (3.5%). The only other *F* ratios to exceed 2.0 were those for the response-location main effect, *F*(1, 43) = 2.74, *p* = .105, \eta _p^2 = .06, and the interaction of response mode and response location, *F*(1, 43) = 3.10, *p* = .085, \eta _p^2 = .07. These reflect a tendency toward a larger advantage for the left response, primarily for finger responses and not hand responses (see Figure 9).

For the left-dominant group, there was only a significant main effect of response mode, *F*(1, 32) = 30.8, *p* < .001, \eta _p^2 = .49, with PE less with the hands (1.1%) than with the fingers (3.5%). The *F* ratios exceeded 2.0 for the two-way interactions of response mode x stimulus location and stimulus location x response location, with the three-way interaction of those variables showing the largest *F* ratio, *F*(1, 32) = 3.50, *p* = .071, \eta _p^2 = .10 (see Figure 9). The effect of response location was significant for hands (0.7% vs. 1.5% for left vs. right), *p* = .024, but not for fingers (3.5% vs. 3.5% for left vs. right).

#### Distribution Analyses

##### Reaction Time

**Figure 10:**
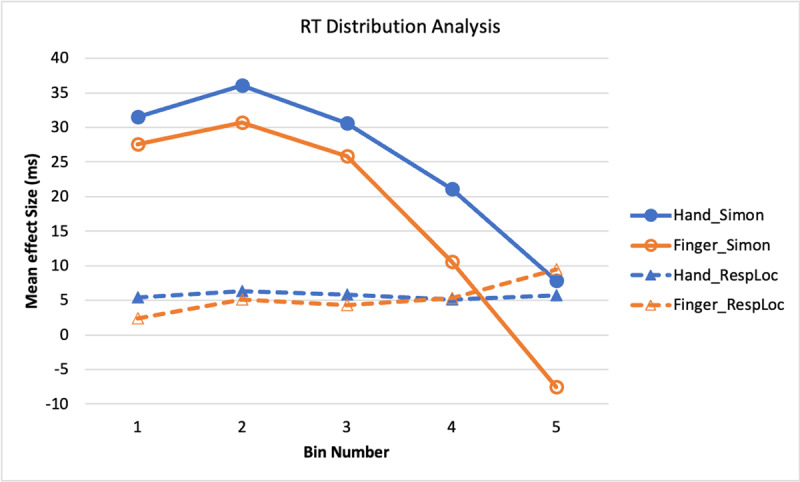
Experiment 2: RT Simon and Response Location Effects as a Function of RT Bin.

RT bin analysis was conducted similar to that in Experiment 1, with error and outlier trials outside [100 ms, 900 ms] being omitted before ranking the trials and dividing them into bins (see Figure 10).

For the Simon effect analysis, the main effect of bin was significant, *F*(1.9, 143.2) = 47.25, *p* < .001, \eta _p^2 = .38 (Huynh-Feldt adjustment applied due to violation of sphericity assumption), and that of response mode was also significant, *F*(1, 76) = 5.66, *p* = .020, \eta _p^2 = .07, with the RT Simon effect being larger with the hands than fingers. Their interaction was not significant, *F*(1.8, 138.2) = 2.08, *p* = .133, \eta _p^2 = .03 (Huynh-Feldt adjustment applied). As for Experiment 1, this result suggests that the difference in hand and finger responses resides mainly in the effectors or apparatus, not in the activation of information for response selection. For the response location effect, none of the variables had a significant effect: response mode, *F* < 1.0; bin, *F* < 1.0; interaction, *F*(2.0, 153.4) = 1.41, *p* = .246, \eta _p^2 = .02 (Huynh-Feldt adjustment applied).

##### Percentage Error

**Figure 11:**
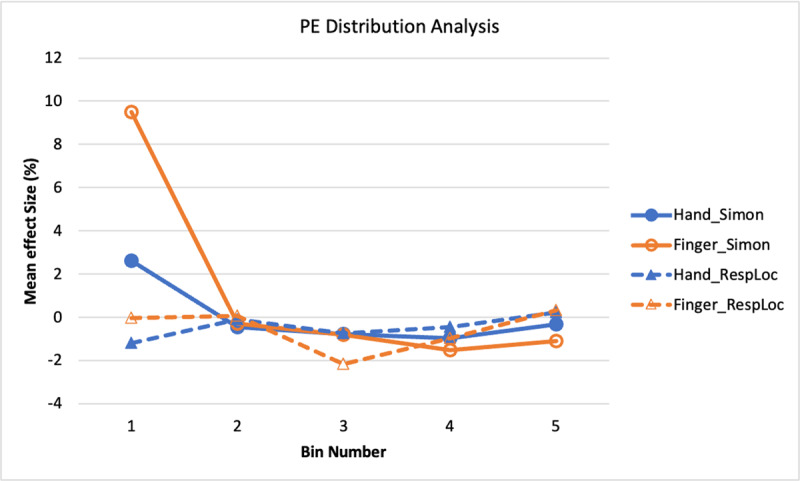
Experiment 2: PE Simon and Response-Location Effects as a Function of RT Bin.

Bin distribution analyses were performed as in Experiment 1, with separate ANOVAs for Simon and response-location effects. The PE data are shown in Figure 11.

For the Simon effect, there was a main effect of bin, *F*(3.1, 231.9) = 29.45, *p* < .001, \eta _p^2 = .28 (Huynh-Feldt adjustment applied due to violation of sphericity assumption), and also a response mode main effect, *F*(1, 76) = 4.79, *p* = .032, \eta _p^2 = .06. Moreover, the interaction of the two variables was significant, *F*(4, 304) = 8.37, *p* < .001, \eta _p^2 = .10. The error Simon effect was primarily restricted to bin 1 (see Figure 11), with the effect at that bin being larger for the finger responses than for the hand responses. For the response-location effect, none of the variables had a significant effect: response mode, *F* < 1.0; bin, *F*(4, 304) = 1.71, *p* = .149, \eta _p^2 = .02; interaction, *F* < 1.0.

### Discussion

The results unambiguously answered the question of whether the hand-press responses yielded less of a Simon effect asymmetry than the finger-press responses. In the RT data, other than a main effect, the only significant interaction involving response mode was the 3-way interaction indicating that the Simon effect was larger with the hand-press responses. The PE data showed the opposite relation of a larger Simon effect for the finger-press responses, suggesting a tradeoff between RT and errors across the two response modes. Because considerable force was required to press down the pedals, it may be that a small percentage of responses began as incorrect but were corrected before a response was recorded. That would account for the higher PE for finger-press responses than for hand-press responses at the first of the five bins of the distribution analysis. Moreover, the changes in the Simon effect across the RT distribution were a similar decreasing function for the hand-press and finger-press responses, implying the same basis for the Simon effects with both response modes.

The Simon effect asymmetry did not differ as a function of response mode, as indicated by there being no significant interactions involving both that variable and response location. The asymmetry results again are in agreement with the effector efficiency account, with the left-handed participants showing an opposite asymmetry than the right-handed participants which did not interact with hand or finger response mode. The difference between dominant and nondominant responses did not vary across the RT distribution, implying a distinct basis from the Simon effect.

## General Discussion

The Simon effect asymmetry refers to the common finding that, with left and right index-finger press responses, the Simon effect is larger for one stimulus location than for the other. For right-handed persons the right stimulus yields the larger effect, whereas for left-handed persons the left stimulus yields the larger effect (Rubichi & Nicoletti, 2006; Seibold et al., 2016; Tagliabue et al., 2007). On the surface, this result pattern suggests that visual processing of the stimulus location corresponding with the dominant hand is faster than that corresponding with the non-dominant hand. However, when the stimulus and response locations are coded separately for analysis, rather than as a single correspondence variable, the asymmetry is revealed to be a response-location main effect (Chen et al., 2021; Seibold et al., 2016; Tagliabue et al., 2007). This latter analysis implies that the asymmetry is not due mainly to the stimulus locations but to the difference in RT for the dominant and non-dominant hands. For the stimulus in the hemispace corresponding with the dominant hand, the RT advantage for the dominant hand increases the calculated Simon effect, whereas it reduces the calculated Simon effect for the hemispace corresponding with the non-dominant hand. In other words, there is no difference in the underlying correspondence effect for the left and right stimulus locations, and the apparent difference in the obtained variables is due to a confounding factor.

According to the effector efficiency hypothesis, the Simon effect asymmetry in RT should not be restricted to left and right index-finger presses. The asymmetry should be found in the form of a response-location main effect when stimulus and response locations are coded separately for any pair of binary responses for which one effector can respond faster than the other. Rubichi and Nicoletti (2006, Experiment 2) showed that the asymmetry favored the dominant hand even when the hands were crossed so that they were located opposite their typical hemispaces. Chen et al. (2021) provided evidence that the asymmetry generalizes to presses of pedals by the left and right feet, for which people show a dominant foot that often matches the hand dominance. Right-dominant persons showed an RT advantage for right foot-press responses, although the foot-press responses appeared to show a bias component that the finger-press responses did not. Experiment 1 of the present study also tested left-dominant persons and had both foot- and hand-press responses made on the same pedals. The left-dominant group showed a Simon effect asymmetry for both the hands and feet, but for foot-press responses the right foot was faster than the left, counter to the effector dominance classification based on foot preference. The right-dominant group did not show an asymmetry for the hand-press responses in Experiment 1, but they did show an effect in Experiment 2 that did not differ significantly from that shown by finger-press responses. Thus, both types of manual responses yielded similar Simon effect asymmetries reflecting faster responding with the right hand.

The delta plot analyses for RT agree with the fact that the Simon effect is due to correspondence of locations, with the difference in effector efficiency having a distinct basis. In both experiments, the Simon effect showed the familiar decreasing function (Proctor et al., 2011) for all response modes (hand presses, foot presses, and finger presses). In contrast, plots of the difference between the non-dominant and dominant effectors yielded relatively flat functions that tended to increase slightly across the RT distribution. Moreover, the difference in error rates between noncorresponding and corresponding trials was large in the first bin of the RT distribution, whereas that between the nondominant and dominant responses was much less pronounced.

Perhaps the most notable finding from the current study is that the foot-press responses for left-dominant persons showed a benefit favoring the right foot. As noted earlier, a similar result was obtained by Peters and Durding (1979) for tapping responses. No definitive conclusion can be reached about why left-dominant persons showed an advantage for right foot press responses. We emphasize, though, that the effector-dominance classification is based on participants selecting the preferred hand with which to carry out verbally described actions. The classification is thus an overall measure that is not tied to actual performance of specific actions. Because motor learning is a critical factor in human motor control (Rosenbaum, 2010; Schmidt et al., 2019), differential experience with the effectors in specific cases may override any preference. The most common form of foot-press control is of a gas pedal in vehicles, for which both left- and right-handed persons must use the right foot. This common experience may account for the right foot being more efficient at making press responses for both left- and right-handers. As one left-dominant person succinctly commented, “Gas pedal is always on the right. I may kick you with my left foot, but my right foot is on the gas” (Reddit, 2022).

The Simon effect occurs not only for irrelevant left-right stimulus locations, as in the present study, but also for the irrelevant words LEFT and RIGHT and left- and right-pointing arrows (Luo & Proctor, 2018, 2020; Miles & Proctor, 2012; Pellicano et al., 2009). If the dominant hand generally responds slightly faster than the non-dominant hand, the asymmetry should be evident not only for the location-based Simon effect but also for the word-based and arrow-based effects. However, if the Simon effect asymmetry is a function of visuospatial processing, it should not occur for the word-based Simon effect. The arrow-based effect would be somewhat less informative since they have both symbolic and visuospatial properties.

In Seibold et al.’s (2016) Experiment 1, participants performed all combinations of Simon tasks that varied in irrelevant stimulus mode (left-right locations or word meanings) and response mode (left-right keypress or vocalizations). As noted in the Introduction, the location-based Simon effect with keypresses showed the Simon effect asymmetry, which interacted with hand dominance. In contrast, the word-based Simon effect with keypresses did not, with all *F* ratios that included response location as a factor being < 1.0. This result implies that difference in efficiency between the dominant and non-dominant effectors is not a general phenomenon. Moreover, neither the stimulus locations nor location words produced a Simon effect asymmetry when the responses were the corresponding names “Left” and “Right”, providing additional evidence that the critical relation is visuospatial correspondence.

Wascher et al. (2001) and Wiegand and Wascher (2005) have argued that in the spatial Simon task, visuomotor facilitation of the corresponding response occurs with the hands in left and right positions that does not occur when stimuli are auditory or the stimuli and responses are positioned along vertical axes. The results of Seibold et al. (2016) suggest that the Simon effect asymmetry, for which the response of the dominant hand is processed more efficiently, may have its basis in visuomotor processing. An additional finding that comports with this possibility is that of Wang et al. (2014). They reported experiments showing that hand proximity (positioning the hands around a computer monitor) produced a larger visual location Simon effect than when the hands (and responses) were more distal. Disregarding issues about the basis for that effect, the relevant finding for present purposes is that they did not find this effect of hand proximity for the word-based Simon effect. So, at least two studies of the Simon effect provided results that are consistent with the idea that potentiation of responses relating to the effectors and their placement is specific to visuospatial processing.

The basic conclusion we have reached from the studies we have run (Chen et al., 2021; Seibold et al., 2016; and the present study) is that the Simon effect asymmetry is not informative as to the cause of the core Simon effect, that is, correspondence phenomena. However, it is instructive as to how the specific features of the task setting affect performance more generally.

## Data Accessibility Statement

Data may be found at https://osf.io/f74qn/.
